# Influenza C in Lancaster, UK, in the winter of 2014–2015

**DOI:** 10.1038/srep46578

**Published:** 2017-04-13

**Authors:** Kate V. Atkinson, Lisa A. Bishop, Glenn Rhodes, Nicolas Salez, Neil R. McEwan, Matthew J. Hegarty, Julie Robey, Nicola Harding, Simon Wetherell, Robert M. Lauder, Roger W. Pickup, Mark Wilkinson, Derek Gatherer

**Affiliations:** 1 Division of Biomedical & Life Sciences, Faculty of Health & Medicine, Lancaster University, Lancaster LA1 4YT, UK; 2 Royal Lancaster Infirmary, Ashton Road, Lancaster LA1 4RP, UK; 3 Centre for Ecology & Hydrology, Lancaster Environment Centre, Lancaster University, Lancaster LA1 4AP, UK; 4 UMR_D 190, Emergence des Pathologies Virales, Aix-Marseille University, 27 Bd Jean Moulin, Marseille cedex 05, 13005, France; 5 Institute of Biological, Environmental & Rural Sciences, Aberystwyth University, Aberystwyth SY23 3DA, UK; 6 Queen Square Medical Practice, 2 Queen Square, Lancaster LA1 1RP, UK

## Abstract

Influenza C is not included in the annual seasonal influenza vaccine, and has historically been regarded as a minor respiratory pathogen. However, recent work has highlighted its potential role as a cause of pneumonia in infants. We performed nasopharyngeal or nasal swabbing and/or serum sampling (*n* = 148) in Lancaster, UK, over the winter of 2014–2015. Using enzyme-linked immunosorbent assay (ELISA), we obtain seropositivity of 77%. By contrast, only 2 individuals, both asymptomatic adults, were influenza C-positive by polymerase chain reaction (PCR). Deep sequencing of nasopharyngeal samples produced partial sequences for 4 genome segments in one of these patients. Bayesian phylogenetic analysis demonstrated that the influenza C genome from this individual is evolutionarily distant to those sampled in recent years and represents a novel genome constellation, indicating that it may be a product of a decades-old reassortment event. Although we find no evidence that influenza C was a significant respiratory pathogen during the winter of 2014–2015 in Lancaster, we confirm previous observations of seropositivity in the majority of the population. (170 words).

## Introduction

### Clinical presentation

Influenza C (family *Orthomyxoviridae*, genus *Influenzavirus C*, species *Influenza C virus*) produces malaise, coryza and fever when administered to susceptible adult volunteers^1^. Historically, influenza C has been regarded as the least serious of the three species of influenza infecting humans, and seasonal vaccination programmes have been confined to influenzas A and B. More recent studies confirmed influenza C’s production of a mild respiratory illness in healthy adults, with only occasional complications^2^.

However, in a paediatric context, acute respiratory illness and/or pneumonia have been reported as a consequence of influenza C infection^3^,^4^,^5^,^6^,^7^,^8^ especially in those under 2 years old^9^, as well as vomiting, diarrhoea, acute otitis media^10^, a high rate of hospitalization^11^ and even acute encephalopathy^12^. This growing awareness of the paediatric clinical importance of influenza C raises the issue of its inclusion in the annual seasonal influenza vaccine, or its position as a candidate for vaccine development specifically for infants.

### Epidemiology

Nearly 40% of adult volunteers were susceptible to administered influenza C^1^. The 60% who did not develop disease is consonant with observation of seropositivity levels of 59% in Spain^13^, 61% in France^14^ and 57% in Brazil^15^, and suggests that seropositivity may possibly confer resistance. By contrast, other studies have suggested that antibodies against influenza C tend to be more universal: 100% in an isolated Philippine village^16^ and in the USA^17^, 90% in Czechoslovakia^18^, 86% in the Soviet Union^18^ and 70% in East Germany^19^. Some studies have also found age-structured variability: in California, seropositivity of 64% in children under 5 but 98% in adults^20^; in Japan, 40–50% in early childhood to nearly 100% in adulthood^21^; in Louisiana, 47% in children to 96% in younger adults, but then a decline to 18% in the over-65s^22^; in France, 46% seropositivity in children, 76% in younger adults, but only 44% in the over-50s^14^.

Influenza C does not appear to be seasonal, based on contemporaneous two-year surveys of its occurrence in Bucharest and Japan from 1988–1990^6^,^23^. Using this observation together with the seropositivity data, it is possible to propose several epidemiological scenarios. The first of these is that influenza C is an endemic virus in human populations, with approximately lifelong immunity conferred by first exposure. The decline in seropositivity in later life^14^,^22^, potentially due to immunosenescence, would then provide the virus with opportunities to infect individuals for a second time. The second scenario is that the virus is only intermittently epidemic, with variation in seropositivity a reflection of previous epidemic history in different locations. The third scenario is that the virus is endemic but antigenically variable over time^24^. Seropositivity would therefore be an unreliable guide to the true immune status of any individual.

### Phylogenetics and molecular evolution

The rate of nucleotide substitution is lower in influenza C than in A and B^25^,^26^,^27^,^28^, and reassortment has been detected^3^,^6^,^25^,^28^,^29^,^30^. There is also evidence of positive selection at two residues in the receptor-binding domain of the haemagglutinin-esterase (HE) protein, but the overall ratio of non-synonymous to synonymous substitutions (omega) across the genome is low, individual proteins ranging from 0.05 to 0.13^28^. The low levels of omega indicate a virus that is well adapted to its host, but the presence of positive selection in the HE receptor-binding domain also indicates selective pressure from the host immune system. This provides a molecular explanation for the observed antigenic drift^24^ and some evidence against the scenario that humans are likely to acquire lifelong immunity.

The issue of endemicity versus sporadic epidemics also remains unresolved. Only one candidate epidemic surge has been identified, in Japan in 2004^4^. The existence of reassorted strains indicates that double infection with two or more strains cannot be very infrequent, implying that it ought to be possible to detect numerous (or at least >1) strains co-circulating both temporally and geographically, previously demonstrated in Japan^4^. Indeed, a continually shifting pattern of segment combinations, referred to as genome constellations^28^, is observed when full genomes are studied, a phenomenon also seen in influenza B^31^. Eight genome constellations circulating in the 1990s differed from the genome constellations present in a set of reference genomes from the 1940s to the 1980s^28^.

## Results

### Participants

Of the 148 participants, 69 were male and 79 female. 71 were symptomatic and 77 asymptomatic. Distribution of male and female participants within symptomatic and asymptomatic groups was assessed by a 2 × 2 chi-square test and was not statistically significant. Except for a relative excess of age group 20–29 participants (mostly from the university), age approximated a normal distribution.

### Influenza C seropositivity

**Figure 1:**
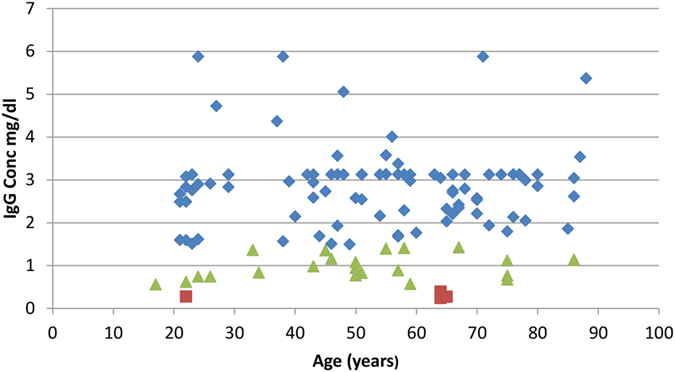
Anti-influenza C IgG concentration (mg/dl), plotted for each individual against age. Blue: >2 standard deviations above negative control; green: 1–2 standard deviations above negative control; red: <1 standard deviation above negative control.

Of the 148 participants, 129 consented to donate serum. Of these 99 were seropositive and 30 negative, giving a figure of 77% seropositivity. Figure 1 shows the anti-influenza C IgG concentration by age. Gender differences in seropositivity were also nearly absent (male mean 2.5 mg/dl, female mean 2.3 mg/dl) with no statistical significance on t-test, but symptomatic individuals had slightly more IgG (symptomatic mean 2.6 mg/dl, asymptomatic mean 2.2 mg/dl), significant on a t-test at p < 0.05. A Mann Whitney U-test was performed on the distribution of seropositive individuals between each age group, and was not statistically significant.

### Detection of viral RNA

Two participants out of 148 (1.4%), aged 51 years and 70 years, both asymptomatic, were detected as positive for influenza C using quantitative PCR, at 135 ng and 160 ng total viral RNA respectively, corresponding to 1.9 × 10^10^ and 2.2 × 10^10^ genome copies. Since influenza viruses are believed to have a single genome copy per virion^32^, this indicates approximately 2 × 10^10^ virions per individual nasopharyngeal swab. On deep sequencing (accessions SRR4733498 and SRR4733494), only one patient showed sufficient levels of influenza C reads for genome assembly to be attempted (SRR4733498).

### Genetic relationships of isolated influenza C genome segments

**Figure 2:**
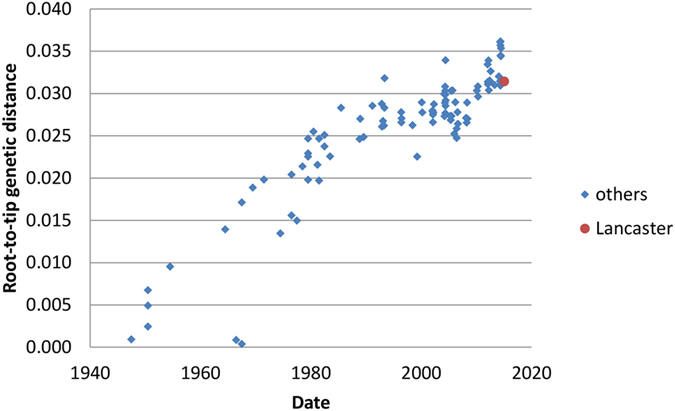
Root-to-tip distance in a neighbour joining tree for segment 1 (encoding PB2) of the influenza C genome. 100 full-length or near full-length genome segments (2365 bases) are used plus the 724 discontinuous bases of segment 1 derived from deep sequencing. C/Lancaster/1/2015 has a degree of divergence from the root consistent with molecular clock-like behaviour in its lineage.

**Figure 3:**
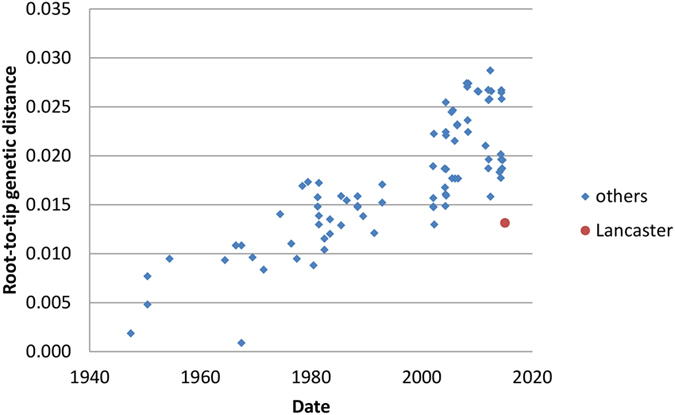
Root-to-tip distance in a neighbour joining tree for segment 6 (encoding M1/CM2) segment of the influenza C genome. 86 full-length or near full-length genome segments (1180 bases) are used plus the 380 discontinuous bases of segment 6 derived from deep sequencing. C/Lancaster/1/2015 is less divergent from the root than it should be given its known sampling date, consistent with a perturbation of molecular clock-like behaviour in its lineage.

Partial genome segment sequences were obtained from deep sequencing for segments 1, 5, 6 and 7, encoding PB2, NP, M1/CM2 and NS1/NS2 respectively. Those greater than 200 bases are deposited in GenBank, accession numbers KY075640 - KY075642. Insufficient reads were available to assemble the other segments. Although breadth of coverage across segments is low (ranging from 22% in segment 5 to 32% in segment 6), there is sufficient genetic information to assign each fragment to a clade as defined previously^28^, using Bayesian phylogenetics. Plotting of the root-to-tip genetic distance on a neighbour-joining tree using TempEst showed that molecular clocks apply best to segments 2 and 7 (PB2 and NS1/NS2), but that both segments 5 and 6 (NP and M1/CM2) have lower root-to-tip distances for C/Lancaster/1/2015 than expected. Figures 2 and 3 shows the TempEst plots for segments 1 and 6 (PB2 and M1/CM2), giving examples of clock-like and non-clock-like behaviour, respectively. The TempEst plots for segments 5 and 7 (NP and NS1/NS2) are Supplementary Figures 3 and 4 respectively. The new strain of influenza C identified was designated C/Lancaster/1/2015.

**Figure 4:**
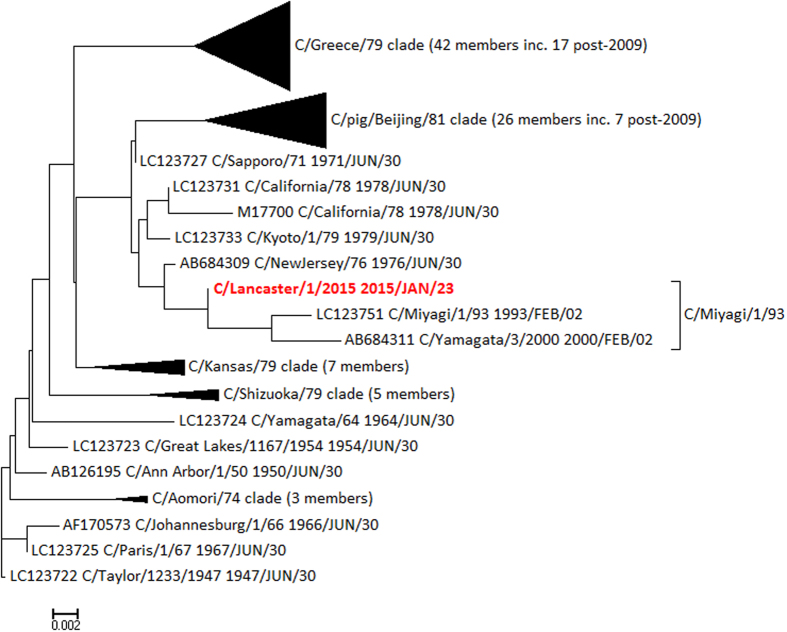
Neighbour joining tree rooted on C/Taylor/1233/1947 for segment 5 (NP), annotated with clades as previously derived^28^, demonstrating the closer relationship of C/Lancaster/1/2015 (red) to NP segments of the C/Miyagi/1/93 clade than to recent isolates. Scale: substitutions per site.

**Figure 5:**
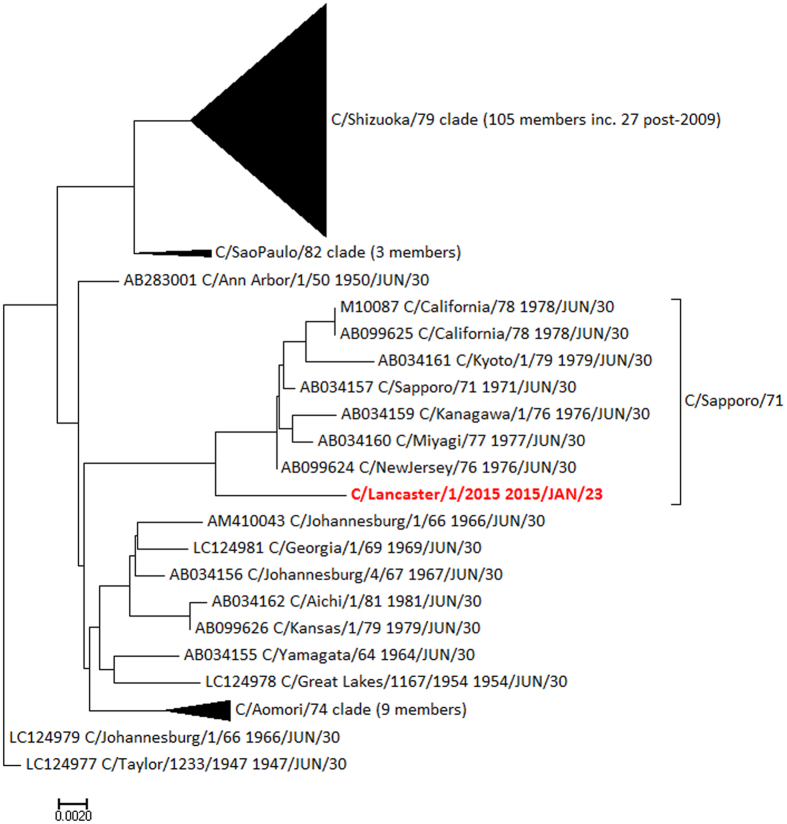
Neighbour joining tree rooted on C/Taylor/1233/1947 for segment 7 (NS1/NS2), annotated with clades as previously derived^28^, demonstrating the closer relationship of C/Lancaster/1/2015 (red) to NS1/NS2 segments of the C/Sapporo/71 clade from the 1970s than to recent isolates. Scale: substitutions per site.

Clade memberships were determined by examination of Bayesian phylogenetic trees produced in BEAST, as previously^28^ and then annotated onto the neighbour-joining trees used for the molecular clock analysis. Figure 4 shows the tree for segment 5 (encoding NP), demonstrating that C/Lancaster/1/2015 belongs to the C/Miyagi/1/93 clade, and not to the C/Greece/79 and C/pig/Beijing/81 clades circulating in recent isolates. Figure 5 shows the tree for segment 7 (encoding NS1/NS2) has an even more distant relationship to recent genomes, being part of the C/Sapporo/71 clade last seen in 1979. The phylogenetic trees for PB2 and MP are given in Supplementary Figures 1 and 2, and further confirm the genetic distance between C/Lancaster/1/2015 and other recently sequenced genomes. Clade memberships are then synthesised to derive the relationship between C/Lancaster/1/2015 and defined genome constellations (Table 1).

## Discussion

Our participant group were 77% seropositive to influenza C. This is slightly higher than the 57–61% levels from studies in western Europe and Brazil^13^,^14^,^15^, within the range of the 70–90% found in eastern Europe^18^,^19^ but still considerably short of those studies reporting universal seropositivity in the USA and east Asia^16^,^17^. As in previous studies, our antibody titre levels were widely variable among those classed as seropositive, and our choice of threshold is purely statistical. However, we also found no statistically significant age-structured or gender-structured variability in seropositivity (Fig. 1). This is at variance with some previous studies in the USA, Japan and Europe^14^,^20^,^21^,^22^. It should also be noted that many serological studies on influenza C are now some decades old and techniques have varied over the years, so individual studies are not necessarily directly comparable. We also cannot exclude the possibility of some cross-reactivity of our influenza C antigen with antibodies to other influenza viruses, but this is also an issue in all previous studies.

Neither of the two participants who were identified as influenza C-positive by PCR generated sufficient deep sequencing reads for complete genomes to be assembled. Our deep sequencing of the nasopharyngeal swabs of both of our PCR-positive participants, produced much fuller genome sequence results for other RNA viruses apart from influenza C, as well as sequences from a range of bacterial species (Atkinson *et al*. in preparation). We therefore do not think that the difficulty in detecting influenza C, or in generating complete genomes, is due to RNA degradation or other technical failure. The 4 segments partially assembled are the least variable segments, but only segment 4, encoding HE, is an outlier in terms of its variability, at 0.042 substitutions per site since 1947, compared to a range of 0.017 to 0.027 for the other segments^28^. Even within HE there are relatively conserved regions within the stalk domain, so we do not believe that failure to assemble HE or other segments is an artefact of excessive stringency in our assembly process.

In the individual with the 4 partial genome segment sequences, it is evident that C/Lancaster/1/2015 is a reassortant that does not fall into any of the genome constellations previously classified^28^ (see also Table 1). It contains a rare NS1/NS2 segment of the C/Sapporo/71 clade, related to sequences that were last observed in the late 1970s. Influenza C genomes sequenced since 2010 all have the C/Shizuoka/79 clade in the NS1/NS2 segment (Fig. 5). C/Lancaster/1/2015 also has a rare NP segment of the C/Miyagi/1/93 clade, related to sequences that were last observed around 2000 (Fig. 4) and typical of genome constellation 4a (Table 1). The other segments are within clades found more recently, although C/Lancaster/1/2015’s position within these clades is never close to any of the recent genome sequences (Supplementary Figures 1 and 2). The exact position of C/Lancaster/1/2015 on each segment’s phylogenetic tree is rarely well supported by Bayesian phylogenetics posterior probability density, but its location within each of the broader clades is well supported. We therefore conclude that its apparent reassortant nature is unlikely to be simply an artefact of partial sequence information.

Tentative reconstruction of the reassortment event may be attempted. Previous work^28^ defines genome constellation 4a as consisting of C/Sapporo/71, C/Miyagi/1/93, C/Sapporo/71 and C/Shizuoka/79 in segments 1, 5, 6 and 7 respectively. The corresponding clades for C/Lancaster/1/2015 are C/Sapporo/71, C/Miyagi/1/93, C/Sapporo/71 and C/Sapporo/71 respectively (Table 1), suggesting that a strain of constellation 4a reassorted with one containing a C/Sapporo/71-clade segment 7. Since no strain containing a segment 7 of this clade has been seen since the 1970s and constellation 4a was only seen in the 1990s, it seems likely that the reassortment event occurred in the 1990s. This would also explain the dissimilarity of C/Lancaster/1/2015 in all of its segments, to other recently sequenced strains. We are tempted to speculate that this reassortant occurred locally in Lancaster, but in the absence of any other British genomes since C/England/892/1983^33^, which is itself incomplete, it is impossible to come a conclusion.

If this scenario is common in small isolated populations, influenza C diversity in terms of shifting genome constellations may be even greater than suggested from the available genomes. The M1/CM2 (Fig. 3) and NP segments (Supplementary Figure 3) for C/Lancaster/1/2015 have lower root-to-tip distances than expected under the assumption of molecular clock-like evolution. When this method is used on database-derived sequences, it is often taken as indicative of incorrect dating. However, given that we know precisely when our samples were collected, it is more likely to reflect a genuinely slower rate of evolution in these samples. The M1/CM2 segment of C/Lancaster/1/2015 is positioned in the phylogenetic tree near segments from the 1980s (Supplementary Figure 1) and the NP segment near segments from the 1990s and 2000 (Fig. 4). This same phenomenon of slowed molecular clock, and aberrant positioning with the phylogenetic tree, has been seen in some strains of Zaire ebolavirus^34^ and also in the 1977 “Russian Flu” H1N1 outbreak^35^, and is thought be a consequence of the virus entering a host population where the serial interval – the time between infection of one host and the next in a transmission chain – is reduced and the virus therefore spends longer in a non-replicative state. For ebolavirus, this is assumed to be a non-typical animal reservoir host, and for Russian Flu possibly a laboratory freezer. Neither of these options would seem to be possible for influenza C, so it may simply be a cumulative result of low transmission rates within relatively small populations slightly delaying the average serial interval, conditions which could apply in Lancaster.

We began this study with the premise that influenza C might be a candidate for inclusion in the seasonal influenza vaccine. Our results do not provide any support for the proposition that vaccination of adults is appropriate, consistent with the conclusion of one other recent report^36^. Although we recruited 71 symptomatic individuals with a range of cold/flu-like symptoms, none of these was influenza C-positive, and none of the respiratory disease burden in Lancaster during our study period can be attributed to influenza C.

There may still be a case for vaccination of children in the light of published reports of serious respiratory disease caused by influenza C in that age group^3^,^4^,^5^,^6^,^7^,^8^,^9^,^10^,^11^,^12^,^37^. We recruited 6 participants in the <9 years age group but none were consented to allow serum sampling. In the single participant in the 10-19 year age group, anti-influenza C IgG levels were at <1 mg/dl and this individual is classified as seronegative (Fig. 1). Haemagglutinin-inhibition assay (HI) would potentially clarify this issue, but in its absence we can only draw limited conclusions at best concerning the clinical implications of seropositivity, as quantified by ELISA.

## Methods

### Ethics

Ethical approval was granted by the UK National Research Ethics Service (NRES), reference 14/LO/1634, Integrated Research Application System (IRAS) Project 147631. The project was registered with the National Institute of Health Research (NIHR), UK as part of the NIHR Clinical Research Network (UKCRN) Portfolio, ID 17799. All methods were carried out in accordance with the relevant guidelines and regulations. Informed consent was obtained from all volunteers of 18 years and older. For those under 18 years, informed parental consent was obtained, together with supervised assent of the volunteer.

### Patient recruitment

Lancaster (54.05°N 2.80°W) is a small city with a population of 45,000 (141,000 including surrounding towns and villages). The permanent resident population is >95% white and 18% are over age 65. Participants were approached in 3 locations from November 2014 to May 2015: 1) Lancaster University, 2) a general practice (GP), 3) hospital clinics. After informed consent was given, patients with coryza and/or other symptoms consistent with respiratory infection, were classified as the symptomatic group (*n* = 71) and the remainder as asymptomatic (*n* = 77). The latter were included to investigate if influenza C could be detected in patients without coryza. Nasopharyngeal (or nasal) swabbing, blood sampling, or both, were performed on the patients, according to consent.

### Sample processing

Nasopharyngeal swabs (MW951SENT, Medical Wire) were used on the rear wall of the nasopharynx or nose (according to consent) of patients, and the tips then snapped off directly into Sigma Virocult® medium.

Blood was drawn or taken from a finger prick, according to consent, using Beckton Dickinson Serum Separator® tubes (SST™). Serum was separated at 1000–2000 g for 10 minutes (for arm samples) or at 6000–15000 g for 90 s (for finger-prick samples) and then stored at −80 °C.

RNA was extracted from the nasopharyngeal swabs using a MagMAX™ Viral RNA Isolation Kit (Ambion). The quality and quantity of RNA extracted from samples was assessed by spectrophotometry using the NanoDrop® 1000 Spectrophotometer V3.3.0 (Thermo Fisher Scientific). cDNA was prepared using a High-Capacity RNA-to-cDNA™ Kit (Applied Biosystems®, Life Technologies™) and a Veriti® Thermal Cycler (Applied Biosystems®, Life Technologies™). The samples were incubated at 37 °C for 60 minutes, before stopping the reaction at 95 °C for 5 minutes and then holding at 4 °C. Once completed, the plates were stored at −20 °C.

Polymerase chain reaction (PCR) was then performed using a 7500 FAST Real-Time PCR system (Applied Biosystems®, Life Technologies™) with thermo-cycling carried out as follows: one cycle of 95 °C for 10 min and 45 cycles of 95 °C for 15 s and 60 °C for 1 min. PCR primers for influenza C were as used previously^38^, and quantification was performed by reference to a positive control sample at 32 ng/μl. Concentrations were converted into genome copy numbers using http://scienceprimer.com/nucleotide-molecular-weight-calculator. Samples judged positive after quantitative PCR were processed using the Illumina Nextera XT library kit and deep sequenced in 2 × 126 bp format using an Illumina HiSeq2500 system.

Enzyme-linked immunosorbent assay (ELISA) was performed on the serum samples using influenza C antigen as previously described, and using the same antigen preparation^38^, with goat anti-human HRP-conjugated secondary antibody (ab6858, Abcam®) and SureBlue™ TMB Microwell Peroxidase Substrate solution. Absorbance was measured at 450 nM using a Wallac Victor2™ (Perkin Elmer) plate reader. Anti-influenza C IgG was quantified by calibration of the peroxidase reaction against a standard dilution series of IgG concentrations. The threshold for seropositivity was placed at 2 standard deviations above the mean level of the negative control serum.

### Genome segment sequence assembly

Illumina reads were trimmed of adapters and other non-genomic elements using CutAdapt 1.1^39^ (https://pypi.python.org/pypi/cutadapt), fastq-mcf 0.11.3^40^ (https://expressionanalysis.github.io/ea-utils), and trim_galore (http://www.bioinformatics.babraham.ac.uk/projects/trim_galore/), within the Read_cleaner pipeline (Gatherer, unpublished). Ethical approval required that no genetic material remain within the samples which could enable identification of patients. Therefore, human genome and transcriptome sequences were removed by iterative alignment onto the NCBI, Ensembl and UCSC human iGenomes (http://support.illumina.com/sequencing/sequencing_software/igenome.html), first using bowtie 1.1.1^41^ (http://bowtie-bio.sourceforge.net/index.shtml), then BWA 0.7.12-r1039^42^ (http://bio-bwa.sourceforge.net) within the Valet pipeline (Gatherer, unpublished). Following each alignment, extraction of unaligned reads was achieved using samtools 0.1.19^43^ (http://samtools.sourceforge.net) and the next alignment commenced. Bowtie, BWA and samtools were co-ordinated using the Vanator pipeline^44^ (https://sourceforge.net/projects/vanator-cvr). The resulting trimmed and cleaned reads are available from the Sequence Read Archive (https://www.ncbi.nlm.nih.gov/sra/: BioSamples SAMN05954290 and SAMN05954291, Runs SRR4733498 and SRR4733494).

Influenza C genome C/Victoria/2/2012 (Genbank ref. KM504282) was selected as a representative of recently circulating influenza C and alignment of cleaned reads carried out using bowtie within the Valet pipeline. Consensus sequences were constructed using samtools 0.1.19 (bcftools and vcfutils functions). C/Victoria/2/2012 was used to fill gaps in the consensi and the bowtie alignment repeated. This cycle was performed until a stable consensus was obtained for each genome segment. The same process was repeated using BWA and combined consensi obtained. Alignment of reads to the final consensi was examined with Tablet^45^ (https://ics.hutton.ac.uk/tablet). Resulting assemblies of more than 200 bases were submitted to GenBank (references KY075640 - KY075642). The new strain of influenza C identified was designated C/Lancaster/1/2015.

### Phylogenetics and genome constellations

Sequence alignments of composite partial segments with full influenza C genomes from GenBank, were performed using Muscle^46^ in MEGA^47^ (http://www.megasoftware.net) and neighbour joining trees^48^ constructed. Clock-like behaviour in sequence evolution on those trees was checked using TempEst^49^ (http://tree.bio.ed.ac.uk/software/tempest). Bayesian phylogenetic analysis was performed in BEAST v.1.8.3^50^ (http://tree.bio.ed.ac.uk/software/beast/). A Tamura 3-parameter (T93 + G) substitution model^51^, coalescent constant size tree prior and relaxed lognormal clock were run for 100 million iterations in BEAST, with a burn-in of 25%. Genome constellations were determined by establishing the clade, as previously defined^28^, in which each genome segment was located.

### Statistical analyses

on volunteers and ELISAs, BAM files and reference genomes for genome assemblies, genome fragments too short for inclusion in GenBank, BEAST inputs and outputs, TempEst inputs and outputs and pipeline Perl scripts, are available from: doi://10.17635/lancaster/researchdata/111.

## Additional Information

**How to cite this article:** Atkinson, K. V. *et al*. Influenza C in Lancaster, UK, in the winter of 2014-2015. *Sci. Rep.* **7**, 46578; doi: 10.1038/srep46578 (2017).

**Publisher's note:** Springer Nature remains neutral with regard to jurisdictional claims in published maps and institutional affiliations.

## Supplementary Material

Supplementary S1

Supplementary S2

Supplementary S3

Supplementary S4

Supplementary Information

## Figures and Tables

**Table 1:** Clade membership of segments of C/Lancaster/1/2015 and the prior clade and genome constellation classifications as previously derived^28^.

Genome segment (encoded protein)	Clade of segment in Lancaster consensus, as defined by Gatherer^28^	Genome constellation in which that clade is present	Clade(s) of other post 2009 genomes
1 (PB2)	C/Sapporo/71	All, except 5	C/Greece/79; C/Sapporo/71
5 (NP)	C/Miyagi/1/93	4a	C/pig/115/Beijing/81; C/Greece/79
6 (M1/CM2)	C/Sapporo/71	All, except 2 & 3	C/Sapporo/71
7 (NS1/NS2)	C/Sapporo/71	None: clade not seen since 1970s	C/Shizuoka/79

The rightmost column lists those clades found in other segments sequenced from 2010 onwards. Segments 1 and 6 of C/Lancaster/1/2015 are outliers within clades found in other recent genomes, but segments 5 and 7 are not.
