# Erratum: Influenza C in Lancaster, UK, in the winter of 2014–2015

**DOI:** 10.1038/srep46815

**Published:** 2017-05-26

**Authors:** Kate V. Atkinson, Lisa A. Bishop, Glenn Rhodes, Nicolas Salez, Neil R. McEwan, Matthew J. Hegarty, Julie Robey, Nicola Harding, Simon Wetherell, Robert M. Lauder, Roger W. Pickup, Mark Wilkinson, Derek Gatherer

Scientific Reports 7: Article number: 4657810.1038/srep46578; published online: 04 13 2017; updated: 05 26 2017

The original version of this Article incorrectly included the following text in the Methods section:

“Statistical analyses on volunteers and ELISAs, BAM files and reference genomes for genome assemblies, genome fragments too short for inclusion in GenBank, BEAST inputs and outputs, TempEst inputs and outputs and pipeline Perl scripts, are available from: doi://10.17635/lancaster/researchdata/111”.

This now appears in a Data Availability section in the PDF and HTML versions of the Article.

