# Supplementary figures and images for: Influenza C in Lancaster, UK, in the winter of 2014–2015
Source: Sci Rep. 2017 Apr 13;7:46578. doi: 10.1038/srep46578 (PMC5390268; doi:10.1038/srep46578)

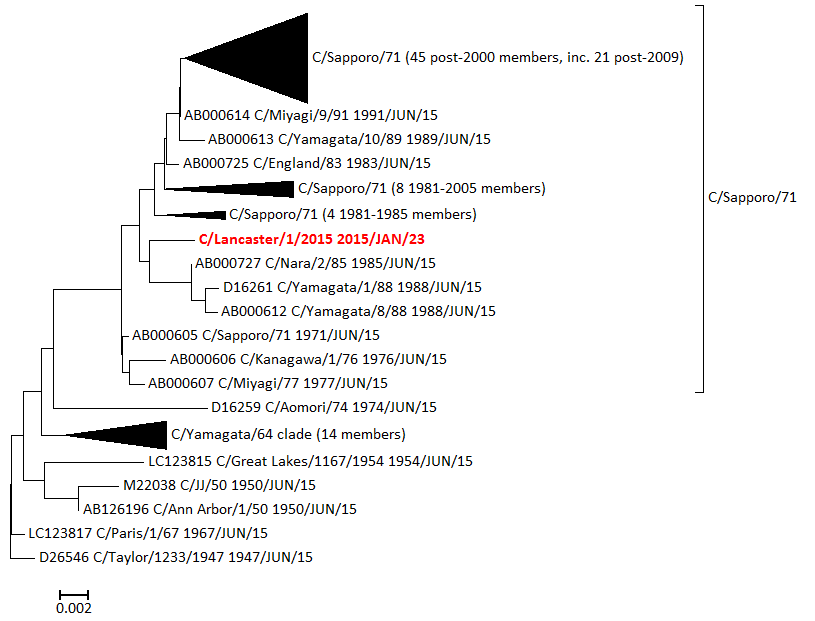

Supplement: Supplementary S1 [file srep46578-s1.tiff]

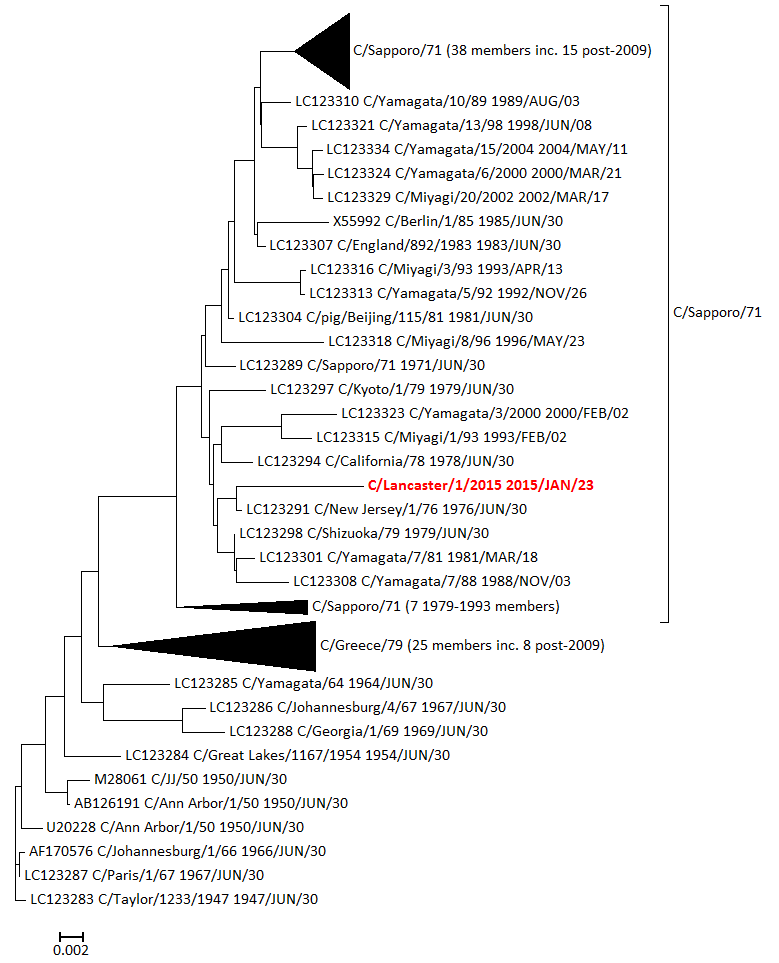

Supplement: Supplementary S2 [file srep46578-s2.tiff]

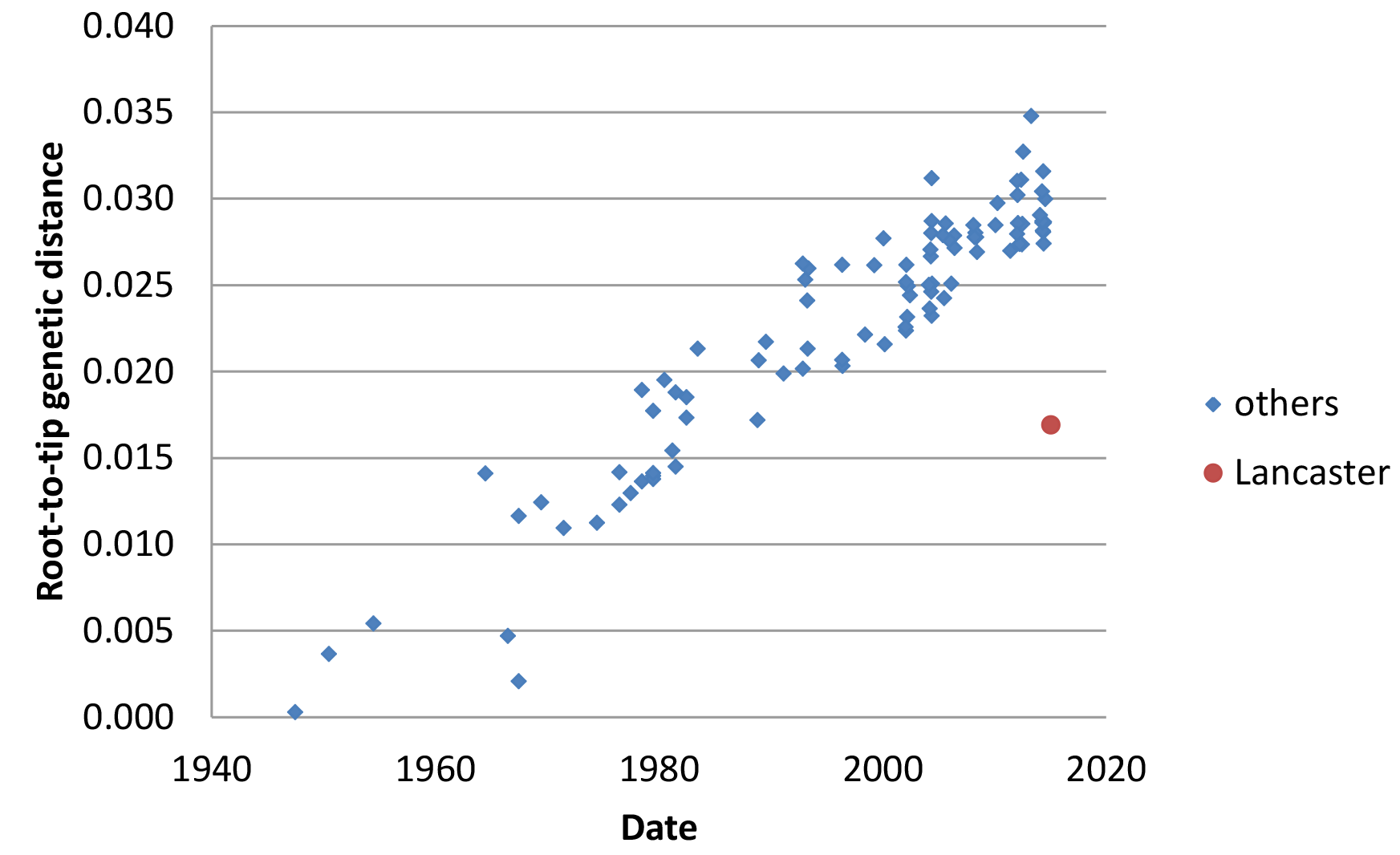

Supplement: Supplementary S3 [file srep46578-s3.tiff]

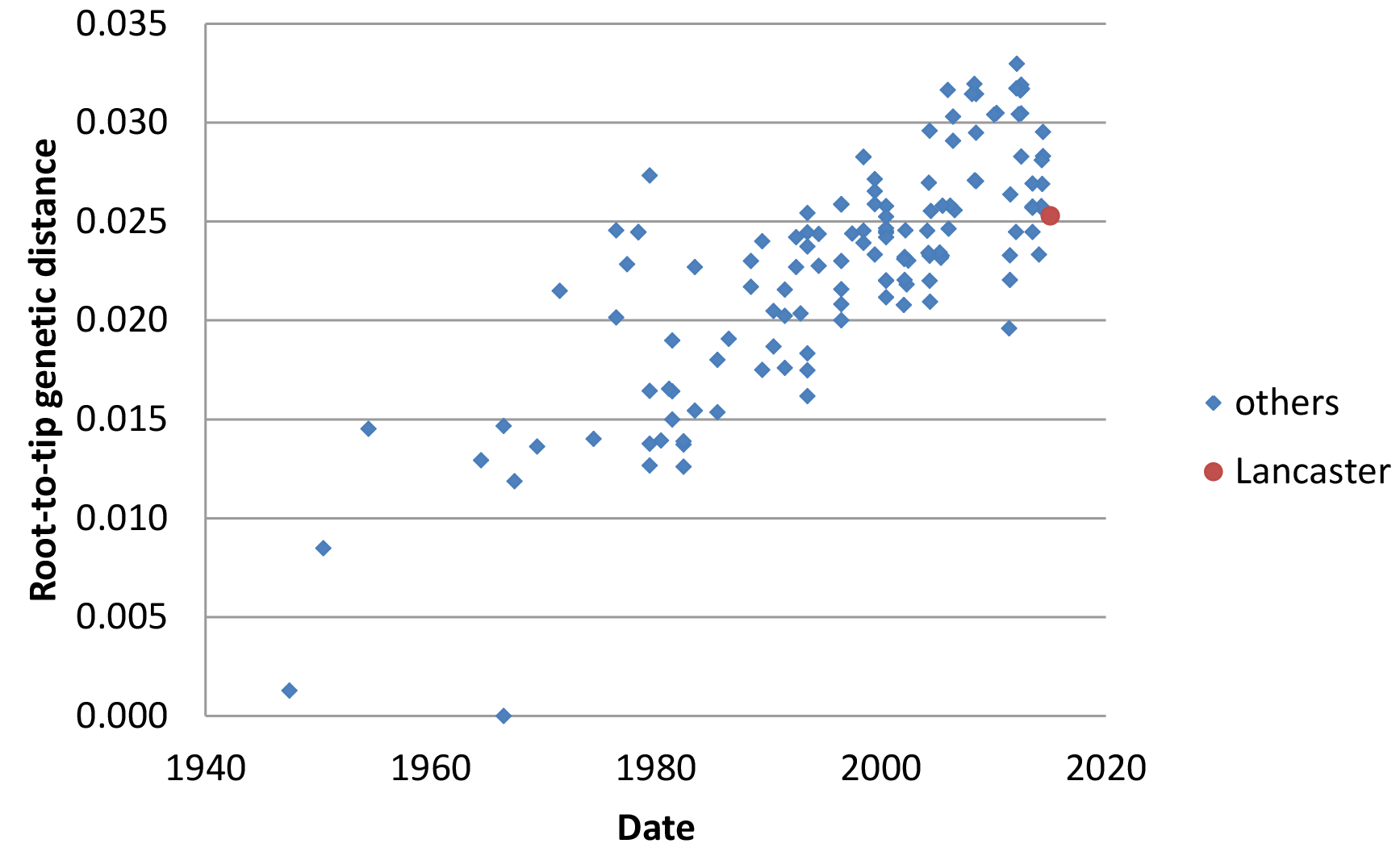

Supplement: Supplementary S4 [file srep46578-s4.tiff]
