# Supplementary material for: Influenza C in Lancaster, UK, in the winter of 2014–2015
Source: Sci Rep. 2017 Apr 13;7:46578. doi: 10.1038/srep46578 (PMC5390268; doi:10.1038/srep46578)
Supplement: Supplementary Information [file srep46578-s5.pdf]

Supplementary figures for:

Influenza C in Lancaster, UK, in the winter of 2014-2015

Kate V Atkinson[1]†, Lisa A Bishop[1,2], Glenn Rhodes[3], Nicolas Salez[4], Neil R McEwan[5], Matthew J Hegarty[5], Julie Robey[6], Nicola Harding[6], Simon Wetherell[6], Robert M Lauder[1], Roger W Pickup[1,3], Mark Wilkinson[2] & Derek Gatherer[1]*

[1] Division of Biomedical & Life Sciences, Faculty of Health & Medicine, Lancaster University, Lancaster LA1 4YT, UK

[2]Royal Lancaster Infirmary, Ashton Road, Lancaster LA1 4RP, UK

[3]Centre for Ecology & Hydrology, Lancaster Environment Centre, Lancaster University, Lancaster LA1 4AP, UK

[4]UMR_D 190, Emergence des Pathologies Virales, Aix-Marseille University, 27 Bd Jean Moulin, 13005 Marseille cedex 05, France

[5]Institute of Biological, Environmental & Rural Sciences, Aberystwyth University, Aberystwyth SY23 3DA, UK

[6]Queen Square Medical Practice, 2 Queen Square, Lancaster LA1 1RP, UK

† *Present address:* University College London Hospitals NHS Foundation Trust, London

* *Contact for correspondence:* Derek Gatherer, email: d.gatherer@lancaster.ac.uk, Tel: +44-1524-592900. Division of Biomedical & Life Sciences, Faculty of Health & Medicine, Lancaster University, Lancaster LA1 4YT, UK

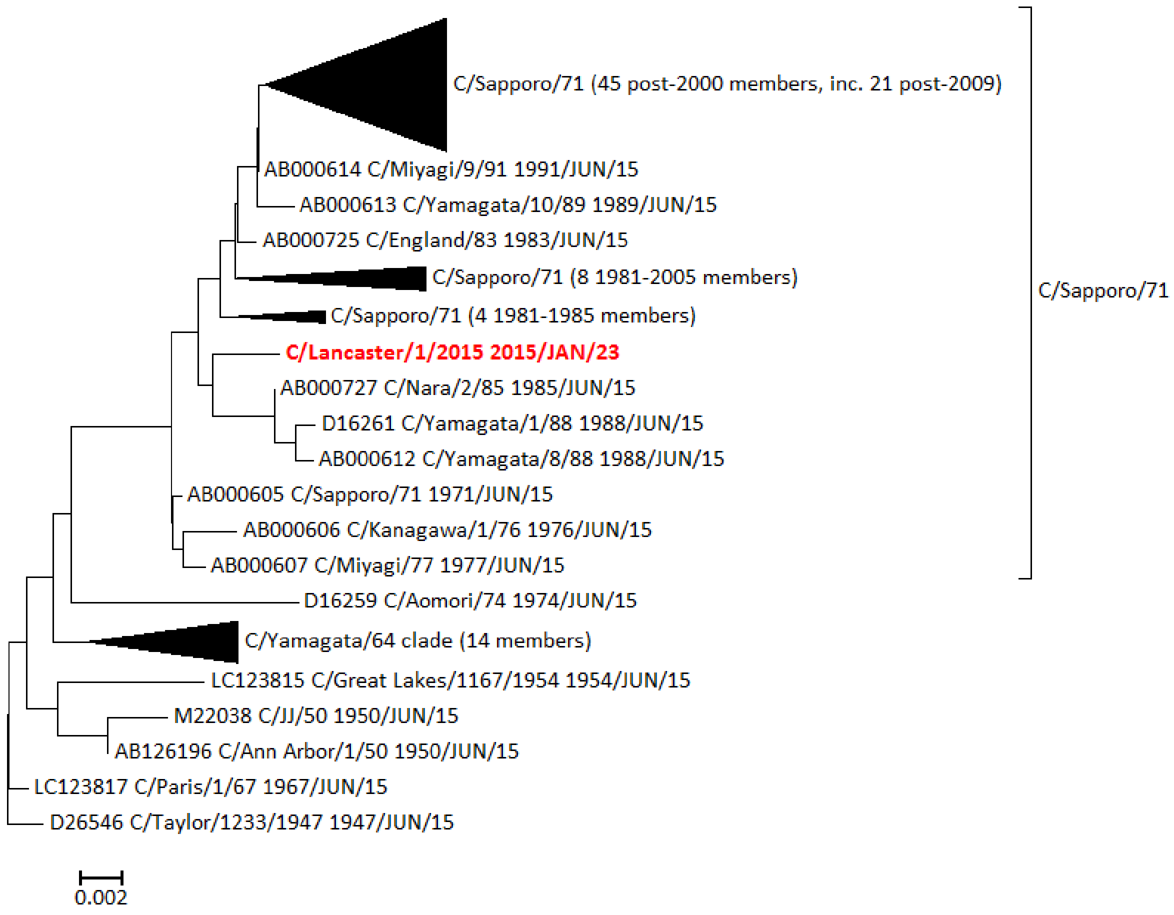

Supplementary Figure 1: Neighbour joining tree rooted on C/Taylor/1233/1947 for segment 6 (M1/CM2), annotated with clades derived from Gatherer [1] and confirmed by BEAST analysis (see Supplementary Data Pack), demonstrating the closer relationship of C/Lancaster/1/2015 (red) to M1/CM2 segments of the C/Sapporo/71 clade from the 1980s than to recent isolates. Scale: substitutions per site.

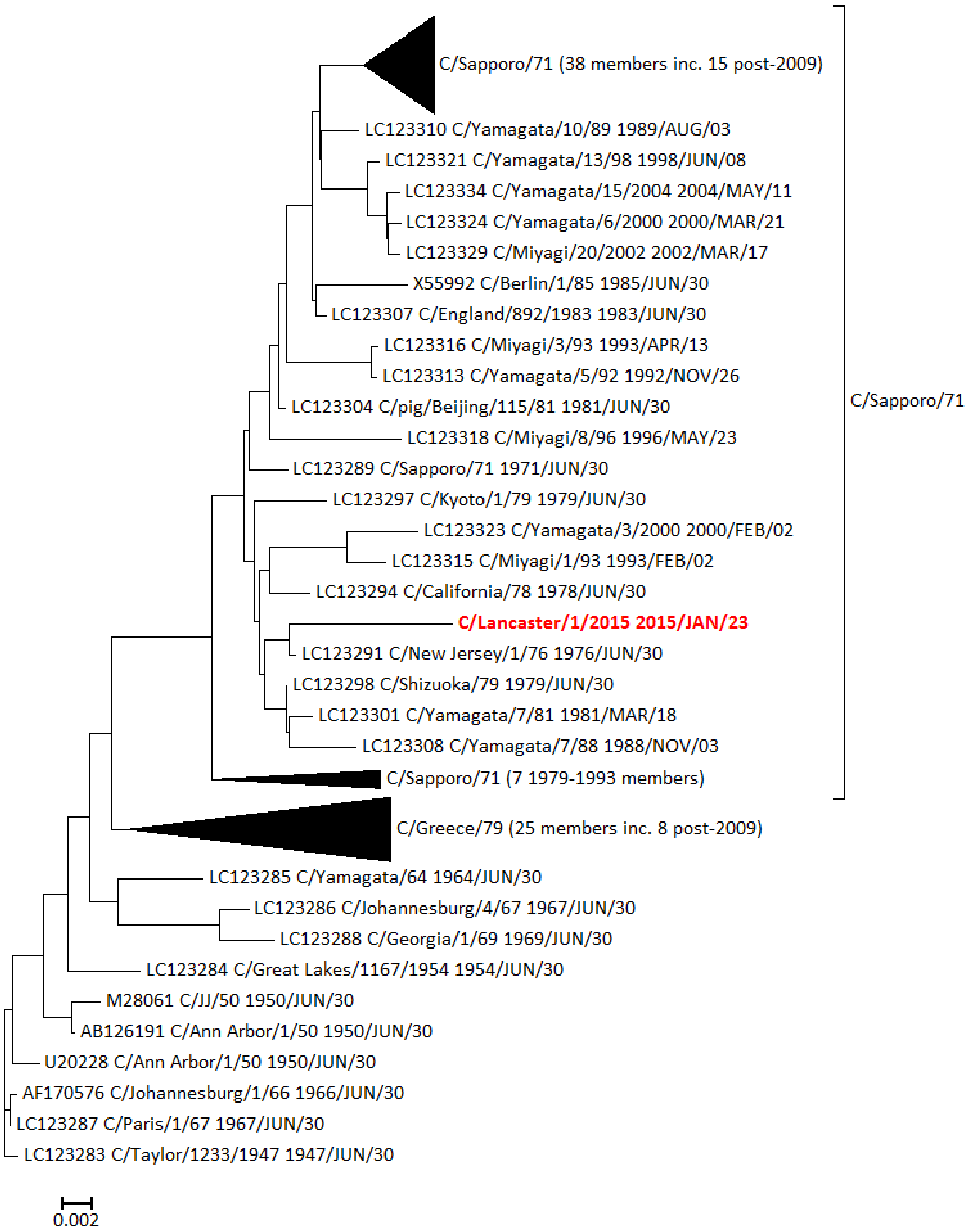

Supplementary Figure 2: Neighbour joining tree rooted on C/Taylor/1233/1947 for segment 1 (PB2), annotated with clades derived from Gatherer [1] and confirmed by BEAST analysis (see Supplementary Data Pack), demonstrating the closer relationship of C/Lancaster/1/2015 (red) to PB2 segments of the C/Sapporo/71 clade from the 1970s and 1980s than to recent isolates. Scale: substitutions per site.

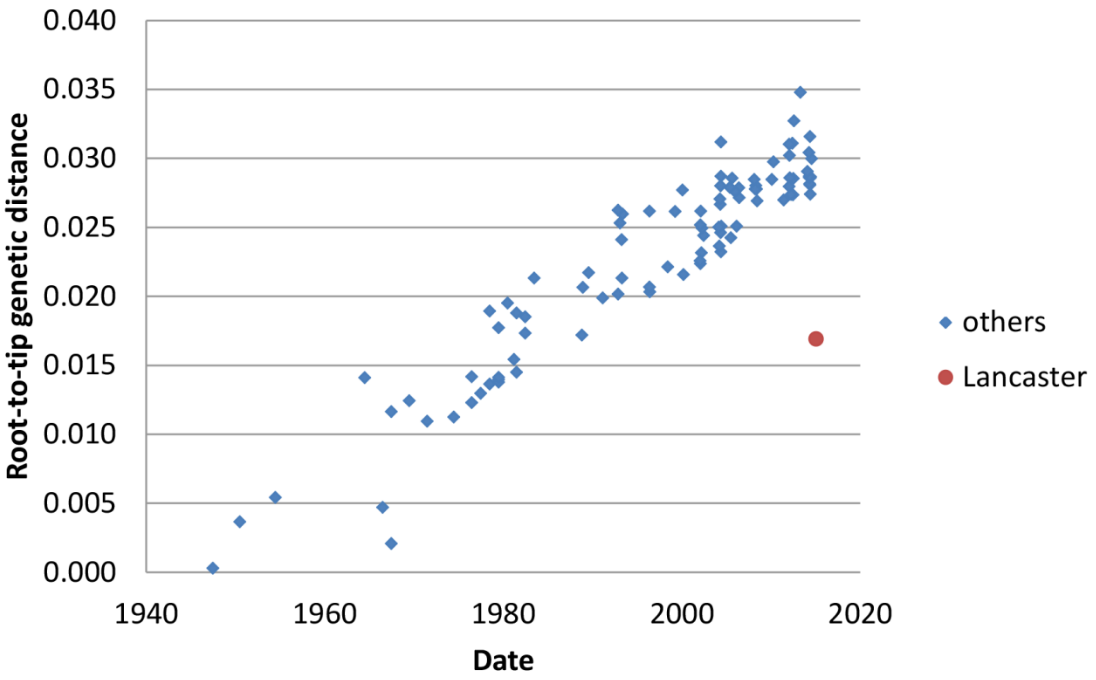

Supplementary Figure 3: Root-to-tip distance in a neighbour joining tree for segment 5 (NP) of the influenza C genome. 96 full-length or near full-length genome segments (1809 bases) are used plus the 397 discontinuous bases of segment 5 derived from deep sequencing. C/Lancaster/1/2015 is less divergent from the root than it should be given its known sampling date, consistent with a perturbation of molecular clock-like behaviour in its lineage.

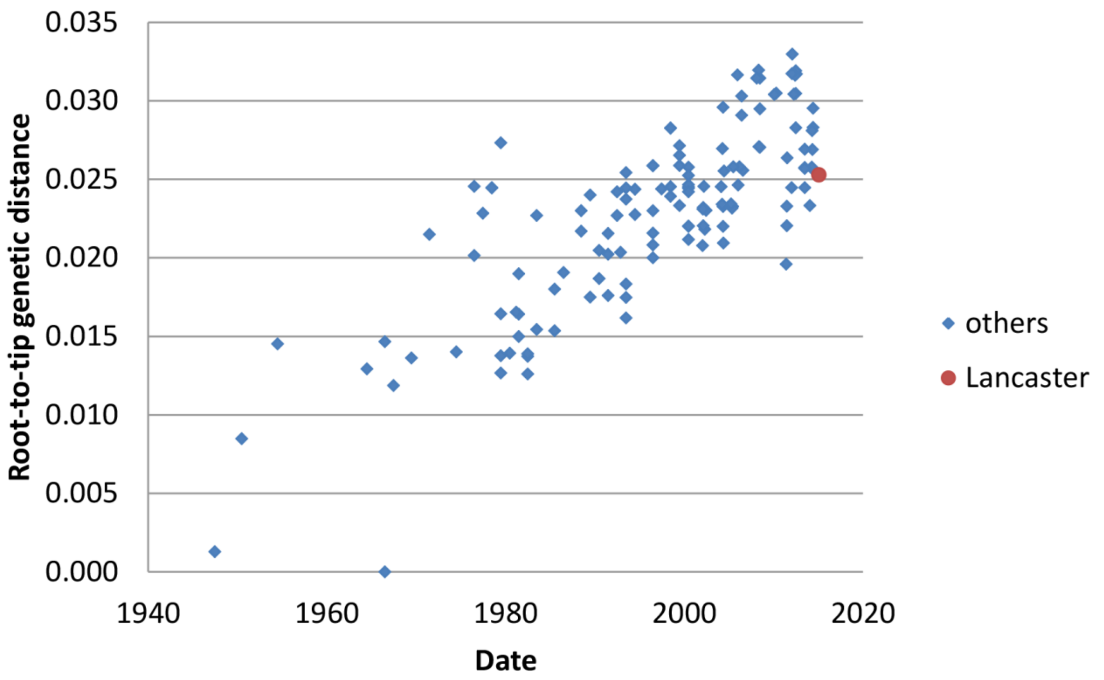

Supplementary Figure 4: Root-to-tip distance in a neighbour joining tree for segment 7 (NS1/NS2) segment of the influenza C genome. 134 full-length or near full-length genome segments (935 bases) are used plus the 288 discontinuous bases of segment 7 derived from deep sequencing. C/Lancaster/1/2015 has a degree of divergence from the root consistent with molecular clock-like behaviour in its lineage.

References

1 Gatherer, D. Tempo and mode in the molecular evolution of influenza C. *PLoS currents* **2**, RRN1199, doi:10.1371/currents.RRN1199 (2010).
